# Chromosome 15q25 (*CHRNA3-CHRNA5*) Variation Impacts Indirectly on Lung Cancer Risk

**DOI:** 10.1371/journal.pone.0019085

**Published:** 2011-04-29

**Authors:** Yufei Wang, Peter Broderick, Athena Matakidou, Timothy Eisen, Richard S. Houlston

**Affiliations:** 1 Section of Cancer Genetics, Institute of Cancer Research, Sutton, Surrey, United Kingdom; 2 Department of Oncology, Cambridge University, Cambridge, United Kingdom; University of Bristol, United Kingdom

## Abstract

Genetic variants at the 15q25 *CHRNA5-CHRNA3* locus have been shown to influence lung cancer risk however there is controversy as to whether variants have a direct carcinogenic effect on lung cancer risk or impact indirectly through smoking behavior. We have performed a detailed analysis of the 15q25 risk variants rs12914385 and rs8042374 with smoking behavior and lung cancer risk in 4,343 lung cancer cases and 1,479 controls from the Genetic Lung Cancer Predisposition Study (GELCAPS). A strong association between rs12914385 and rs8042374, and lung cancer risk was shown, odds ratios (OR) were 1.44, (95% confidence interval (CI): 1.29–1.62, *P* = 3.69×10^−10^) and 1.35 (95% CI: 1.18–1.55, *P* = 9.99×10^−6^) respectively. Each copy of risk alleles at rs12914385 and rs8042374 was associated with increased cigarette consumption of 1.0 and 0.9 cigarettes per day (CPD) (*P* = 5.18×10^−5^ and *P* = 5.65×10^−3^). These genetically determined modest differences in smoking behavior can be shown to be sufficient to account for the 15q25 association with lung cancer risk. To further verify the indirect effect of 15q25 on the risk, we restricted our analysis of lung cancer risk to never-smokers and conducted a meta-analysis of previously published studies of lung cancer risk in never-smokers. Never-smoker studies published in English were ascertained from PubMed stipulating - lung cancer, risk, genome-wide association, candidate genes. Our study and five previously published studies provided data on 2,405 never-smoker lung cancer cases and 7,622 controls. In the pooled analysis no association has been found between the 15q25 variation and lung cancer risk (OR = 1.09, 95% CI: 0.94–1.28). This study affirms the 15q25 association with smoking and is consistent with an indirect link between genotype and lung cancer risk.

## Introduction

An association between common variants in the *CHRNA5-CHRNA3-CHRNB4* nicotinic acetylcholine receptor subunit gene cluster on chromosome 15q25 and lung cancer risk has recently been reported [1], [2], [3]; notably with the single nucleotide polymorphism (SNP) rs1051730 and highly correlated SNPs (including rs12914385). This association was first identified directly through genome-wide association (GWA) studies of lung cancer conducted by Amos et al [1] and Hung et al [2]. Concurrently with publication of these two studies Thorgeirsson et al [3] reported a statistically significant association with the same 15q25 variants and metrics of nicotine dependence, concluding that this explained the elevated risk of lung cancer they also observed. Prior to these studies the 15q25 SNP rs16969968 which is correlated with rs1051730 was identified through candidate gene studies as a determinant of nicotine dependence [4]. The association between the 15q25 locus tagged by rs16969968/rs1051730 and other correlated SNPs has been robustly replicated for smoking related traits including, cigarettes per day (CPD) and heavy smoking, in both candidate gene studies [5] and recent large meta-analyses of GWA data [6], [7], [8].

While the lung cancer risk associated with 15q25 variants reported in the various studies are comparable, relative risk ∼1.3, researchers differ as to whether the association is direct or simply reflective of propensity to smoke and hence increased exposure to tobacco carcinogens. It has been argued that the association with CPD is not sufficient to explain the association between 15q25 variation and lung cancer risk [9], suggesting a direct role for 15q25 in lung cancer development. This possibility is supported by the finding of an increased risk of lung cancer in both ever- and never-smokers associated with 15q25 risk variants reported by Hung et al [2]. The observation of higher lung cancer risks in lower smoking-exposed strata and in individuals with a family history of the disease has also been interpreted to implicate 15q25 variants in both smoking behaviour and directly in lung cancer [10]. Support for this assertion comes from the finding that lung cancer risks associated with 15q25 variants have been reported to be essentially unchanged after adjusting for CPD [9]. Other studies have, however failed to demonstrate a lung cancer association in never-smokers raising doubt about a direct effect of the 15q25 locus on disease risk [1], [10], [11].

Extensive genotyping of the 15q25 region has recently provided evidence for multiple association signals defining nicotine dependence within this chromosome region; specifically a second probable disease locus independent of rs1051730–rs16969968, which is annotated by the highly correlated SNPs rs8042374, rs6495309 and rs578776 [5], [6], [12].

To further explore the relationship between 15q25 variation and lung cancer risk, specifically evidence for an indirect effect, we have conducted a detailed analysis of the relationship between the two 15q25 risk loci with smoking phenotype quantifying impact of variants on lung cancer. To provide increased power to demonstrate a relationship between 15q25 genotype and lung cancer risk in never-smokers, we conducted a meta-analysis, pooling our study findings with previously published data.

## Materials and Methods

The flow diagram for this study and supporting PRISMA checklist are available as supporting information; see Checklist S1 and Diagram S1.

### Ethics Statement

Collection of samples and clinico-pathological information was undertaken with informed and written consent and in accordance with the tenets of the Declaration of Helsinki. Ethical review board approval was obtained from the Royal Marsden NHS Hospitals Trust and the UK Multicentre Ethics Committee.

### Study participants and SNP genotyping

To evaluate 15q25 variation on the risk of lung cancer we derived rs12914385 and rs8042374 genotypes from our previously reported GWA study of lung cancer which annotated the two independent loci at 15q25 [12], [13]. rs12914385 is highly correlated with both rs16969968 and rs1051730 (*D'* = 1.0, r^2^ = 0.81, and *D'* = 1.0, r^2^ = 0.83, respectively based on HapMap CEU) thus annotating the same locus. Comprehensive details of our GWA study have been previously reported [12], [13]. Briefly, a series of 4,343 lung cancer cases (2,782 male; mean age at diagnosis 66 years) were ascertained through the Genetic Lung Cancer Predisposition Study (GELCAPS) [14]. All of the cases had pathologically confirmed lung cancer. For controls we genotyped 1,479 healthy subjects (461 male; mean age at sampling 63 years) ascertained from GELCAPS. Detailed smoking quantity data was available on 4,019 cases and 907 controls. We defined smokers in both cases and controls on the basis of having had a lifetime exposure of more than 100 cigarettes. Family history of lung cancer in cases was based on the definition of having at least one first-degree relative affected with lung cancer. Both cases and controls were British residents and self-reported to be of European Ancestry. Genotyping was conducted using Illumina Human550 BeadChips and Illumina Infinium arrays according to the manufacturer's protocols as previously described [12], [13]. To ensure quality of genotyping, a series of duplicate samples were included and cases and controls were genotyped in the same batches. We have previously confirmed an absence of systematic genetic differences between cases and controls and shown no evidence of population stratification in these sample sets [12], [13].

### Statistical analysis

The risk of lung cancer associated with SNP genotype was assessed by ORs and *P*-values derived from Cochran-Armitage test using logistic regression. Deviation of the genotype frequencies in the controls from those expected under Hardy-Weinberg Equilibrium (HWE) was assessed by the χ^2^ test. To examine the impact of genotype on smoking behaviour, we tested trend in cigarette consumption which has been assessed by log transformed CPD, smoking initiation, cessation and duration using Cochran-Armitage test. To explore the possibility that genotype influences the age of onset of lung cancer we conducted Cochran-Armitage test on average age of diagnosis across genotype strata in both smokers and never-smokers. Age, sex and smoking were adjusted in all the tests when appropriate. When adjusted for, smoking quantity and duration were introduced using the optimal transformation derived by Box-Cox method.

The population attributable risk (PAR), which quantifies the proportion of the total risk of lung cancer which is due to the genetic effect of that locus was estimated using the formulae: 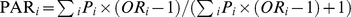, where 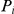 is the prevalence in controls of the lung cancer risk allele at the 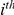 locus, and 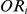 is the OR of the risk allele at the 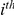 locus.

We estimated the familial relative risk of lung cancer attributable to the smoking behaviour using previously published methodology [15].

All the statistical analyses were undertaken in R (v2.8) software. In all statistical analyses we considered a two-sided *P*-value of 0.05 or less to be statistically significant.

### Meta-analysis

#### Study identification

To identify previously published studies reporting the relationship between 15q25 variation and lung cancer risk in never-smokers we interrogated the electronic database PubMed (from January 1996 up to the end of July 2010). The search strategy included the keywords “lung cancer, risk, genome-wide association, candidate genes”. We searched for any additional studies in the bibliographies of identified publications, including previous review articles.

#### Selection criteria

Studies were eligible if they were based on unrelated individuals and examined the association between lung cancer and polymorphic genotype at 15q25 in never smokers. Only studies published as full-length articles or letters in peer-reviewed journals in English were included in the analysis.

#### Data extraction

Data for analyses, including study design, sample size, ethnicity, as well as allele and genotype frequencies, were extracted from the published articles and summarized in a consistent manner to aid comparison. When a study reported results on different sub-populations according to ethnicity, we considered each sub-population as a separate study in the meta-analysis.

#### Statistical analysis

Raw data of genotype frequencies of 15q25 variant rs16969968 and its proxies, were used for calculation of the study-specific estimates of OR and CI. Meta-analysis was performed under both fixed and random effects models, estimating Cochran's Q statistic to test for heterogeneity and the I^2^ statistic to quantify the proportion of the total variation between studies [16], [17]. To address between-study heterogeneity we derived a pooled odds ratio under a random effects model [16]. An estimate of the potential publication bias was conducted by examination of funnel plots. An asymmetric plot is reflective of publication bias. The funnel plot symmetry was assessed by Egger's test based on inverse-variance weighted regression of the standardized effect size (OR/standard error (SE) of OR) on their precision (1/SE) to test whether the intercept deviates significantly from zero. The significance of the pooled OR was determined by the Z-test and *P*<0.05 was considered as statistically significant.

## Results

The characteristics of lung cancer patients and control series studied are detailed in Table 1. In keeping with the established relationship between smoking and lung cancer risk, the lung cancer cases reported statistically significantly higher rates of CPD and pack years (*P* = 2.55×10^−23^ and *P* = 2.00×10^−13^, respectively). Furthermore, there was however a strong trend in CPD across age groups; cases in the uppermost quantile having a cigarette consumption of 3.2 CPD less than those in the lowest age quantile (*P* = 3.01×10^−21^).

**Table 1:** Details of the lung cancer patients and healthy controls from GELCAPS.

	Cases	Control subjects
Number (Male; %)	4,343 (2,782, 64%)	1,479 (461, 31%)
Mean age (SD) years	65.4 (9.8)	63.0 (10.0)
Family history of lung cancer*	598 (14%)	-
Lung cancer histology		
NSCLC	3312 (76%)	-
SCLC	1027 (24%)	-
Other	2 (<1%)	-
Smoking status		
Never	241 (6%)	553 (37%)
Former	2883 (66%)	585 (40%)
Current	1219 (28%)	341 (23%)
Mean CPD (SD)	23 (19)	18 (11)
Mean pack years (SD)	45 (31)	19 (23)

*defined as having at least one first-degree relative affected with lung cancer.

NSCLC, non-small cell lung cancer;

SCLC, small cell lung cancer.

CPD, cigarettes per day.

RAF, risk allele frequency.

Genotypes were obtained for >95% of cases and controls for rs12914385 and rs8042374 (Table 2). There was no evidence of any systematic bias in genotyping and there was a complete concordance of SNP genotypes between duplicate samples. The allele frequency of each SNP in controls was similar to previously published data on the Northern European population (HapMap, CEU population). Furthermore, there was no evidence of population stratification as the genotype distribution in both control series for each of the SNPs satisfied HWE.

**Table 2:** Risk estimates for rs12914385 and rs8042374 stratified by selected variables.

	Lung cancer cases	Control subjects	Crude OR (95% CI)	*P*-value	Adjusted OR (95% CI)	*P*-value
**rs12914385**	n (%)	n (%)				
Smokers§						
CC	1230 (30.6)	373 (41.1)	1.00 (Ref)			
TC	1973 (49.1)	413 (45.5)	1.45 (1.24–1.70)	3.74×10^−06^	1.47 (1.25–1.73)	4.08×10^−06^
TT†	815 (20.3)	121 (13.3)	2.04 (1.63–2.55)	3.67×10^−10^	2.00 (1.59–2.52)	3.99×10^−09^
*RAF, per allele OR*	0.45	0.36	1.43 (1.29–1.59)	2.10×10^−11^	1.43 (1.28–1.59)	1.79×10^−10^
Familial cases¥						
CC	154 (26.3)	373 (41.1)	1.00 (Ref)			
TC	292 (49.9)	413 (45.5)	1.71 (1.35–2.18)	1.14×10^−05^	1.71 (1.33–2.19)	2.30×10^−05^
TT†	139 (23.8)	121 (13.3)	2.78 (2.05–3.78)	7.03×10^−11^	2.81 (2.04–3.87)	2.76×10^−10^
*RAF, per allele OR*	0.49	0.36	1.67 (1.44–1.95)	2.37×10^−11^	1.68 (1.44–1.96)	9.41×10^−11^
Never-smokers						
CC	100 (41.8)	217(39.2)	1.00 (Ref)		1.00 (Ref)	
TC	109 (45.6)	260 (47.0)	0.91 (0.66–1.26)	0.57	0.90 (0.64–1.27)	0.56
TT†	30 (12.6)	76 (13.7)	0.86 (0.53–1.39)	0.53	0.81 (0.49–1.34)	0.41
*RAF, per allele OR*	0.35	0.37	1.09 (0.87–1.36)	0.47	1.10 (0.87–1.39)	0.43
**rs8042374**						
Smokers§						
AA†	2668 (66.5)	541 (59.6)	1.00 (Ref)			
AG	1207 (30.1)	314 (34.6)	0.78 (0.67–0.91)	1.60×10^−03^	0.78 (0.66–0.92)	2.51×10^−03^
GG	136 (3.4)	52 (5.7)	0.53 (0.38–0.74)	1.86×10^−04^	0.52 (0.37–0.73)	2.01×10^−04^
*RAF, per allele OR*	0.82	0.77	1.32 (1.17–1.50)	8.19×10^−06^	1.33 (1.17–1.51)	1.44×10^−05^
Familial cases¥						
AA†	413 (70.7)	541 (59.6)	1.00 (Ref)			
AG	155 (26.5)	314 (34.6)	0.65 (0.51–0.81)	2.18×10^−04^	0.63 (0.50–0.80)	1.84×10^−04^
GG	16 (2.7)	52 (5.7)	0.40 (0.23–0.72)	1.95×10^−03^	0.41 (0.23–0.74)	3.05×10^−03^
*RAF, per allele OR*	0.84	0.77	1.56 (1.29–1.88)	5.05×10^−06^	1.57 (1.29–1.92)	7.21×10^−06^
Never-smokers						
AA†	135 (56.5)	314 (56.8)	1.00 (Ref)		1.00 (Ref)	
AG	86 (35.98)	206 (37.3)	0.97 (0.70–1.34)	0.86	1.01 (0.73–1.41)	0.94
GG	18 (7.5)	33 (6.0)	1.27 (0.69–2.33)	0.44	1.37 (0.73–2.59)	0.33
*RAF, per allele OR*	0.75	0.75	1.05 (0.82–1.34)	0.7	1.10 (0.85–1.42)	0.47

RAF, risk allele frequency.

RAF of rs12914385 and rs8042374 in the 1958 Birth Cohort 0.39 and 0.76 respectively.

§ Current and former smokers combined.

† Risk genotype.

¥ excludes 6 of the never-smoker cases which reported a family history of lung cancer.

Adjusted for age, sex and categorized smoking quantity CPD (0–10; 11–20; 21–30; 31+).

### Impact of 15q25 genotype on smoking and lung cancer in smokers

Both SNPs showed a statistically significant association with lung cancer risk in a strong dose-dependent fashion in smokers (OR = 1.43 and 1.32 respectively; Table 2). These associations remained statistically significant after adjustment for age, sex and categorized CPD (Table 2). On the basis of the risk associated with each of the variants ∼30% of the PAR of lung cancer is underscored by the 15q25 variation in smokers.

There was a significant 1.2-fold over-representation of rs12914385 and rs8042374 risk alleles amongst lung cancer cases which had reported a family history of lung cancer (Table 2; *P*-values for case-only analysis, 0.004 and 0.03 respectively).

We examined the relationship between genotype and smoking behavior firstly considering CPD as a quantitative trait (Table 3). A strong correlation between cigarette consumption and risk genotype at both 15q25 loci was observed in cases (Table 3). While a similar relationship between smoking and genotype was shown in controls it was not statistically significant (Table 3). This is likely to simply reflect small sample size and hence limited power to demonstrate a relationship, as while we had >90% power to demonstrate a relationship between 15q25 genotype and CPD (1 CPD per risk allele) in cases, for controls power was only ∼50% stipulating a *P*-value of 0.05. In cases those homozygous for rs12914385 and rs8042374 risk alleles smoked on average 2.0–2.3 CPD more than individuals homozygous for non-risk alleles (Table 3); the corresponding impact of rs12914385 and rs8042374 genotype on CPD in the cases was 0.9–1.0 per allele (Data not shown). Secondly, we examined the relationship between genotype and heavy smoking, defined as >20 CPD. In the lung cancer cases a strong relationship between SNP risk genotype and heavy cigarette consumption was shown (Table 4).

**Table 3:** Smoking intensity and dependence by 15q25 genotype.

	Lung cancer cases			Control subjects		
Genotype	n	Mean CPD	*P*-value*	n	Mean CPD	*P*-value*
rs12914385						
CC	1230	21.6		373	18.1	
TC	1973	22.2		413	18.5	
TT†	815	23.9		121	18.5	
			4.76×10^−05^			0.38
rs8042374						
AA†	2668	22.7		541	18.6	
AG	1207	21.8		314	18.0	
GG	136	20.7		52	17.9	
			7.55×10^−03^			0.48

*From Kruskal-Wallis test.

†Risk genotype.

**Table 4:** Prevalence of rs12914385 and rs8042374 risk alleles in light and heavy smoking lung cancer cases and controls.

		Lung cancer cases			Control subjects		
Genotype	CPD	n	RAF	*P-value* *	n	RAF	*P-value* *
rs12914385							
	1–20	2611	0.43		688	0.36	
	21+	1407	0.47	5.65×10^−04^	219	0.37	0.58
rs8042374							
	1–20	2606	0.81		688	0.76	
	21+	1405	0.83	3.72×10^−03^	219	0.79	0.37

*P values calculated using the Cochran-Armitage test.

RAF, risk allele frequency.

We determined if smoking initiation or cessation was modified by 15q25 genotype, among ever-smokers. We observed no statistically significant association between genotype at either locus with smoking initiation in cases alone or controls alone or in combined subjects among ever-smokers. Similarly we found no evidence that genotype modified age of cessation among former-smokers (data not shown).

We then examined the possibility that genotype might influence age of onset of lung cancer. Armitage trend test was used to detect trend in mean age of onset across genotype groups. For rs12914385 risk genotype, homozygous carriers had a mean age of diagnosis of 64.7 years compared with 65.7 and 66.4 years in heterozygote and wild-type genotype carriers respectively (*P* = 0.0001). Corresponding mean ages at diagnosis by rs8042374 genotype were 65.5, 66.4 and 66.8 years, respectively (*P* = 0.003). These differences remained statistically significant after correction for sex and CPD and duration of smoking using linear modeling. The median smoking duration for carriers of risk alleles at rs12914385 or rs8042374 was 1 year higher than that of non-carriers (44 vs 43 years and 43 vs 42 years, respectively), albeit non-significant.

### Impact of 15q25 genotype on lung cancer in never-smokers

In never-smokers comparison of genotype frequencies in cases and controls provided no evidence that lung cancer risk is influenced by either rs12914385 or rs8042374 genotype (Table 2). We examined the possibility that 15q25 genotype might influence age of onset of lung cancer in never-smokers. For rs12914385 risk genotype, homozygous carriers had a mean age of diagnosis of 65.8 years compared with 65.6 and 66.0 years in heterozygote and wild-type genotype carriers respectively (*P* = 0.99). Corresponding mean ages at diagnosis by rs8042374 genotype were 66.8, 64.1 and 66.2 years, respectively (*P* = 0.31).

**Figure 1:**
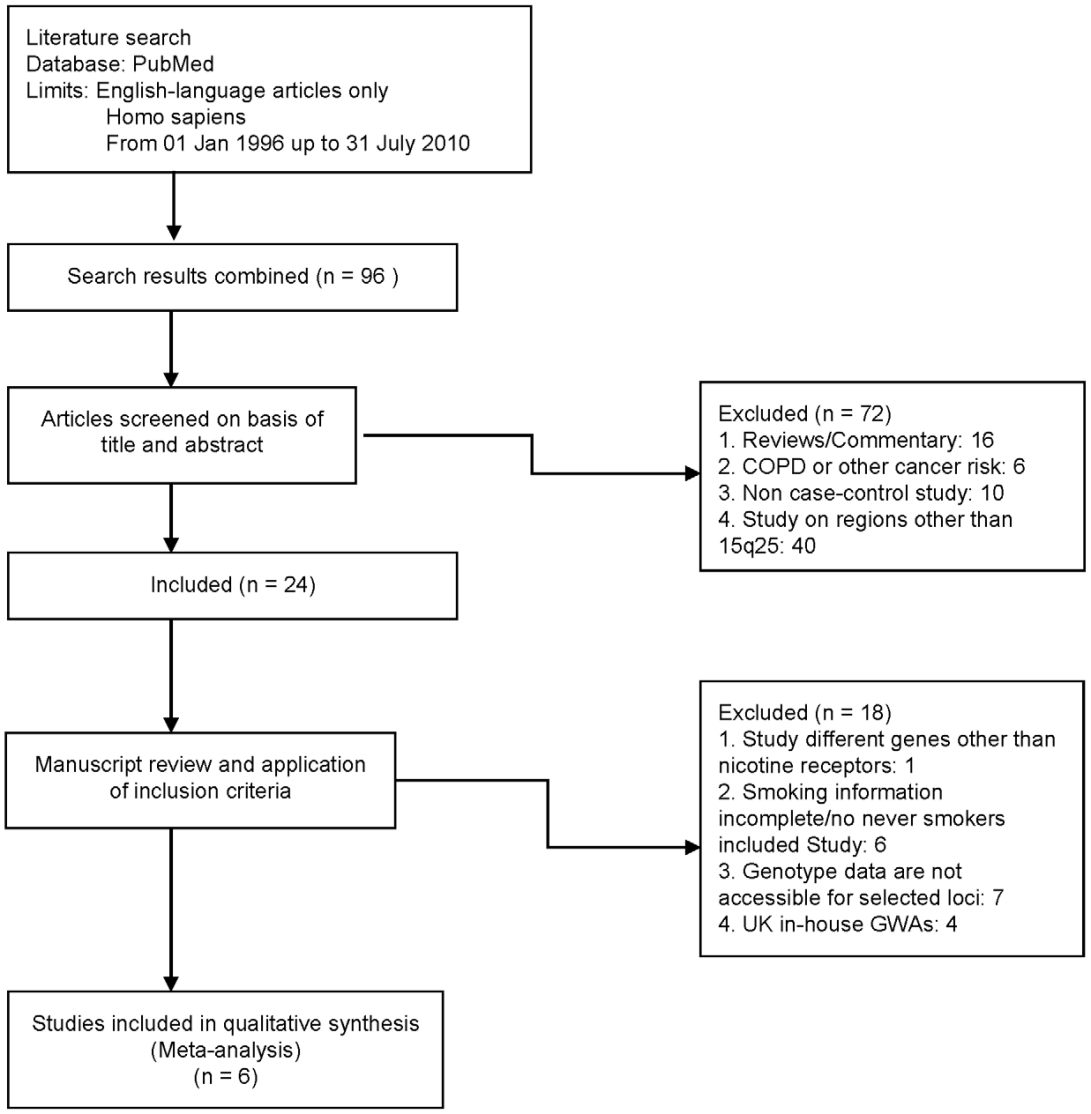
Inclusion and exclusion criteria for studies.

**Figure 2:**
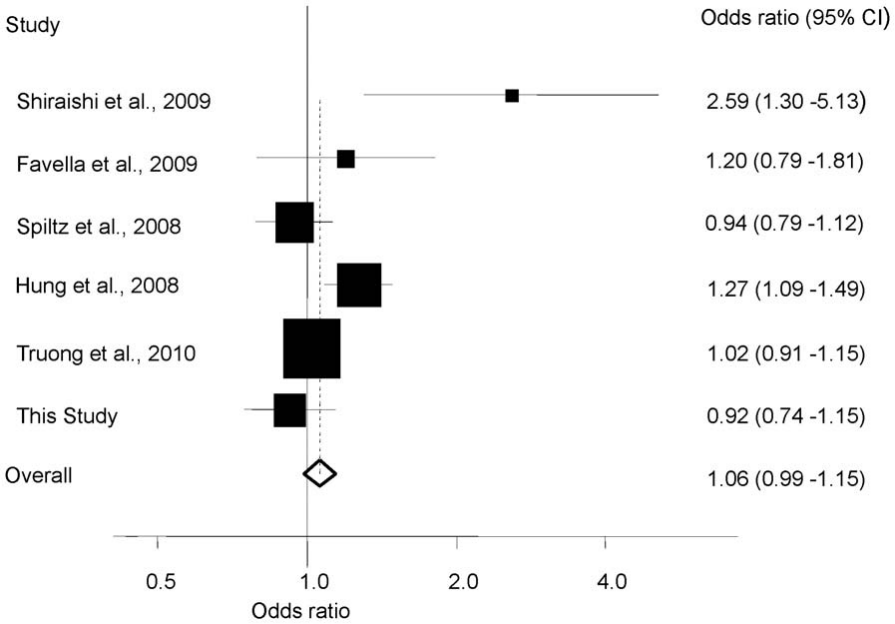
Forest plots of odds ratios for lung cancer in never-smokers associated with the 15q25 locus. Boxes represent OR point estimates, their areas being proportional to the inverse variance weight of the estimate. Horizontal lines represent 95% confidence intervals. Diamond (and broken line) represents the summary OR computed under a fixed effects model, with 95% confidence interval given by its width. The unbroken vertical line is at the null value (OR = 1.0).

To maximize the possibility of identifying an association between 15q25 genotype and lung cancer in never-smokers we conducted a meta-analysis pooling our study with previously published case-control studies. We retrieved 95 studies using our search criteria (Figure 1). Five of these 95 studies met our pre-determined criteria for inclusion; two were based on Caucasians [10], [11], one on the Japanese population [18], and two reported case-control studies from multiple countries [2], [19]. The data presented by Amos et al [1] is superseded by the current study and was therefore not analysed. The SNPs rs16969968 and rs1051730 were each genotyped in five of the published studies and rs8034191 in one. As rs12914385, rs1051730 and rs8034191 are highly correlated with rs16969968 (*D*' = 0.98–1.00 and r^2^ = 0.81–0.98, based on HapMap CEU) each SNP can be considered as proxies for another. While the minor allele frequencies of rs1051730 and rs16969968 are lower in Japanese (0.013, 0.013) than in Caucasians (0.35, 0.35), the haplotype defined by the risk SNP alleles is associates with lung cancer risk in the Japanese population [18]. Given the strong correlations between SNP genotypes we therefore considered rs8034191, rs1051730, rs16969968 and rs12914385 as defining a single genetic locus and conducted a meta-analysis of the six studies on this basis. Collectively these five studies and our study provided data on a total of 2,405 never-smoker lung cancer cases and 7,622 controls. Meta-analysis of these six studies provided no evidence for a statistically significant association between 15q25 genotype and lung cancer risk in never-smokers; OR = 1.06 (95% CI: 0.99–1.15, *P* = 0.12) (Figure 2). There was, however between-study heterogeneity (*P_het_* = 0.008, I^2^ = 68%), and the pooled OR under a random effects model was 1.09 (95% CI: 0.94–1.28, *P* = 0.26). Between-study heterogeneity was largely attributable to inclusion of the Japanese study. Omitting this study from the analysis between-study heterogeneity was non-significant but the association remained non-significant with a pooled OR of 1.05 (95% CI: 0.97–1.13, *P* = 0.20; *P_het_* = 0.06, I^2^ = 56%). No publication bias was found by examining either the funnel plot or formal Egger's test (*P* = 0.34).

## Discussion

While an association between 15q25 variation and lung cancer risk is now well established there is currently no consensus on the relative impact of variants on propensity to smoke versus a direct carcinogenic effect. In this study we have conducted a detailed analysis of 15q25 variants, smoking behaviour and lung cancer risk in a population-based series of lung cancer cases to gain insight into the underlying basis of this cancer association.

We confirm previous observations of a strong relationship between the two 15q25 loci annotated by rs12914385 and rs8042374 with both smoking behaviour and risk of lung cancer. We found that for each locus smokers carrying two copies of the risk allele smoked on average two more CPD than those homozygous for non-risk alleles.

Much of the assertion that 15q25 variation has a direct effect on lung cancer risk rather than solely being a proxy for smoking comes from the observation that the lung cancer association is not accounted for by the relationship with smoking quantity. Applying the Doll and Peto model [20] of the dose-response relationship between smoking and lung cancer for those aged 16–25 smoking <40 CPD, Brennan and co-workers estimated that a 1.2 difference in CPD between rs16969968 homozygotes only results in a 9% increase in lung cancer risk [9]. This is substantially lower than the observed association between rs16969968 and lung cancer risk. While we found that adjustment for CPD had little effect on the estimation of lung cancer risk associated with 15q25 variants, it has consistently been shown that for both men and women the number of years of cigarette smoking is far more important than CPD in predicting lung cancer risk [20], [21]. In the Peto and Doll model the risk of lung cancer for men aged 40–79 years is proportional to (CPD+6)^2^.(age-22.5)^4.5^ [20]. Under this model even a 1.0 CPD difference will account for the observed difference in lung cancer risk if genotype influences the duration of smoking by 1 year over a 30 year period. It has been previously shown that *CHRNA3-CHRNA5* variants influence early tobacco addiction [22] and recent studies have demonstrated that 15q25 genotype influences the ability to stop smoking [23]. Hence it is likely that carriers of 15q25 risk genotypes will smoke more consistently over a longer period and have more sustained smoking behaviour. Although we found no strong relationship between duration of smoking and genotype in lung cancer cases our study findings are concordant with this hypothesis.

An over-representation of 15q25 risk alleles in familial lung cancer cases and association with early-onset disease has been suggested to provide evidence for a direct effect of variants on lung cancer risk. It is, however well established that smoking behaviour has a high heritability (0.5–0.7) [24]. Since the relative risk of lung cancer associated with smoking is ∼30 [25] the familial lung cancer risk directly attributable to inherited propensity to smoke is ∼1.4 if 10%–25% of the population consistently smoke. As with the 15q25 locus any over-representation of variants in familial or early-onset lung cancer can readily be accounted for through an indirect mechanism. It is noteworthy in this respect that while 15q25 risk variants were associated with early-onset disease in smokers no such association was seen in never-smoker lung cancer cases. Moreover given that the familial relative risk of lung cancer is ∼1.7 [26], genetically determined smoking behaviour is likely to contribute significantly to the observed clustering of lung cancer.

The design of our study is very similar to the other case-control studies which have previously investigated the relationship between polymorphic variation and lung cancer risk. While data on lung cancer diagnoses are derived from histological records, details on smoking behaviour was obtained through self-administered questionnaires; thus there is a qualitative difference in the robustness of these two endpoints used in our analysis. Self-reported data about smoking behaviours several decades ago is inherently problematic. Cigarette use has been shown to be commonly under-reported by smokers in studies which have correlated self-reported cigarette use with cotinine levels [27]. If underreporting smoking habit or cigarette consumption differs between cases and controls this is a potential source of significant bias in establishing a direct association between the 15q25 locus and lung cancer risk. This is especially of concern as 15q25 genotype influences smoking behaviour. Beside these issues it has been shown that carriers of risk variants extract a greater carcinogenic nitrosamine per cigarette dose [28]. While self-reported CPD has enabled an association between 15q25 and smoking to be demonstrated, even if accurately assessed CPD does not adequately take into account carcinogenic load. In view of this, simple adjustments using self-reported CPD metrics is likely to be suboptimal for teasing out direct effects on lung cancer risk and it is perhaps not surprising that effect sizes for many 15q25 lung cancer associations appear relatively unchanged when simple adjustments are made. Future epidemiological studies seeking to demonstrate a direct effect of 15q25 on lung cancer risk should take into consideration the significant potential issue of confounding in study design.

The strongest epidemiological data supporting a direct role of genetic variation at 15q25 as a risk factor for lung cancer would be provided by demonstration of an association in never-smokers. While our own study of never-smokers was relatively small it had ∼80% power to demonstrate a lung cancer association assuming an OR of 1.3. While this effect size is comparable to that seen for the lung cancer association in smokers it can be asserted that any direct association may be more modest. However, the meta-analysis we conducted failed to demonstrate a significant relationship despite having in excess of 80% power to show a relative risk of 1.1. Hence if there is a direct effect of 15q25 on lung cancer risk it is likely to be overshadowed by the indirect effect.

Although our data thus favours an indirect effect of 15q25 variation on lung cancer risk we cannot entirely exclude the possibility of direct effect. The acid test proving a direct effect is likely to be reliant on biological assays. Evidence that nicotine is either carcinogenic or co-carcinogenic or functions as a tumour promoter for lung cancer would support the plausibility of a direct relationship between the 15q25 locus and lung cancer risk. There is evidence that variants in the region are associated with decreased expression of *CHRNA5* in the lungs and that *CHRNA5* expression is higher in lung cancers favouring a direct role [29]. While the SNP rs16969968 is a non-synonymous SNP causing the D398N substitution in *CHRNA5* and while 398N causes decreased response to a nicotine agonist [30], a direct role of this variant in lung cancer biology has thus far not been shown. Moreover to date data on the direct effect of nicotine on lung cancer biology is sparse and inconsistent (reviewed in [31]).

In conclusion the results of our analyses reaffirm the strong relationship between the 15q25 locus and both smoking and lung cancer risk. However, our findings do not provide evidence for direct effect of 15q25 on lung cancer risk and it is possible to explain this association in smokers through the influence on smoking behaviour. Assertion of a direct effect of variants on lung cancer risk is currently weak and this should not detract from concerted efforts to reduce lung cancer burden through public health initiatives to reduce smoking. Given that 15q25 variation influences smoking behaviour it is possible that assaying 15q25 genotype may have healthcare utility in helping the tailoring of smoking cessation strategies.

### URLs

The R suite can be found at http://www.r-project.org/

HAPMAP: http://www.hapmap.org/

## Supporting Information

Checklist S1 **PRISMA checklist.**
(DOC)

Diagram S1 **PRISMA flow diagram.**
(DOC)
