# Supplementary material for: Chromosome 15q25 (CHRNA3-CHRNA5) Variation Impacts Indirectly on Lung Cancer Risk
Source: PLoS One. 2011 Apr 29;6(4):e19085. doi: 10.1371/journal.pone.0019085 (PMC3084737; doi:10.1371/journal.pone.0019085)
Supplement: Diagram S1 — PRISMA flow diagram. (DOC) [file pone.0019085.s002.doc]

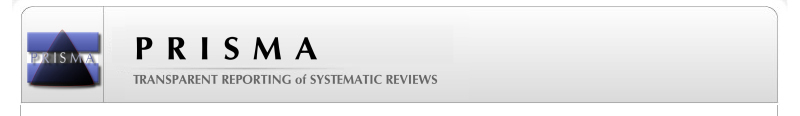
**PRISMA 2009 Flow Diagram**

**Screening**

**Included**

**Eligibility**

**Identification**

Literature search

Database: PubMed

Limits: English-language articles only

Homo sapiens

From 01 Jan 1996 up to 31 July 2010

Articles screened on basis of title and abstract

Excluded (n = 72)

1. Reviews/Commentary: 16

2. COPD or other cancer risk: 6

3. Non case-control study: 10
4. Study on regions other than 15q25: 40

Manuscript review and application of inclusion criteria

Excluded (n = 18)

1. Study different genes other than nicotine receptors: 1
2. Smoking information incomplete/no never smokers included Study: 6

3. Genotype data are not accessible for selected loci: 7

4. UK in-house GWAs: 4

Studies included in qualitative synthesis

(Meta-analysis)
(n = 6)

Search results combined (n = 96 )

Included (n = 24)
